# Physical health assessment quality improvement project

**DOI:** 10.1192/bjo.2021.544

**Published:** 2021-06-18

**Authors:** Maggie Lambert

**Affiliations:** CAMHS, Northern Health Centre

## Abstract

**Aims:**

My aim was to ensure at least 60% of clients in the Acute Day Unit have a ‘physical screening tool’ entry.

**Background:**

As a GP starting training in psychiatry I am very aware of the importance of physical health and the overlap between physical health and mental health. It has been found that there is a 20 year mortality gap for men and 15 year mortality gap for women in people with mental health problems. Thorncroft described this as ‘the scandal of premature mortality’.

Nice Guidelines state: ‘Reducing premature mortality by improving physical healthcare for people with severe mental illness remains an NHS England priority. Funding has been made available to ensure that at least 60% of people who have severe mental illness receive NICE-recommended physical assessments and follow up from 2018/19 onwards.’

The Acute Day Unit seemed to be the ideal situation to try to address this problem as clients are with us for 6-8 weeks during which time their physical health as well as their mental health can be optimised.

**Method:**

I emailed the whole team to invite ideas and questions regarding the QI project and discussed it further at the MDT meeting. It was important at the start to get the whole team on board. Having discussed it we decided to put six blocks of thirty minute slots weekly into the timetable for physical assessments. These were to be booked in by the client's care coordinator. I also added a column onto our team spreadsheet to input whether or not the physical assessment had been done. Frequent encouragements and reminders were sent round the team of which clients still needed a physical assessment.

**Result:**

Before the changes were made 25% of clients were having their physical assessments done. After the changes were made 63% of clients had their physical assessment done, three of the twenty seven clients having only started at the day unit that week.

**Conclusion:**

Having made a change to the system of scheduling six regular slots for physical assessments there has been a dramatic rise in the number of clients having their physical assessment done. As this change has been to the system and will be continued automatically on the team calendar the improvement has been more easily sustained. We are keen to keep improving on this change with an ideal level of over 75% of clients having a physical health assessment.

